# Healthy aging and intergenerational solidarity - Latin America and its moment of opportunity

**Published:** 2012-06-30

**Authors:** Diego A Bernardini-Zambrini

**Affiliations:** Pan American Health Organization (PAHO/WHO)

In recent years, health has seen changes in its global dynamics. The historical stage opened as of the emergence of globalization radically modified two constitutive aspects of human societies from their first expressions: time and space. Crucial aspects that organizations of human beings have taken into account to define their own agendas, fulfill their objectives, and meet their needs. The changes generated by the economic, social, and cultural globalization have revealed the close association among the development of societies, management of public policies, foreign policies of nations, and the interaction with the other players in the global scenario. In this international arena, aspects and effects on the health of individuals can be considered as one of the central themes addressed by contemporary societies.

Currently, the regional health agenda is marked by five phenomena, five themes intimately related to the beginning of the 21^st^century: urbanization, aging, non-transmissible disease, climate change, and migrations. Of these, aging will particularly be a substantive condition for welfare development in our Region in the mid- to long-term future.

In line with this global phenomenon, the World Health Organization has elected Healthy Aging^1^ as central theme for the celebration of the World Health Day in 2012. In Europe, additionally, 2012 has been declared the European Year for Active Aging and Intergenerational Solidarity^2^.

## Why the great significance of the aging process of our society for healthcare?

According to the United Nations, in 2006 Latin America and the Caribbean had over 55 million people over 60 years of age. It is estimated that by 2025 this population segment will increase to 100 million^3^ and by 2050 these will be 200 million . The consequences of this dramatic increase could be quite significant for regional social development, especially regarding policies of social protection, which include healthcare and welfare systems. 

Let's put it this way; life expectancy has increased due to progress and development in issues of health and sanitation. The increase in the size of the cohorts of older adults is showing us new epidemiological disease patterns. The emergence of chronic, non-transmissible diseases and the importance of the social determinants of health as conditions of the life course show us that age is not always accompanied by good health. This will lead to an increase of individuals with loss of autonomy of their daily activities and, thus, a greater need for social assistance and care - formal and not remunerated, with an economic cost for social protection that currently constitutes a true challenge; all within a scenario where the number and proportion of individuals is bigger than ever before and whose expectation is to increase. 

This sequence, course, or pattern of social evolution raises important questions, some of these sensitive for the medical community: is aging a medical or social issue? Is it a problem that must be addressed by geriatricians, family physicians, or by the whole team of healthcare professionals? Are medical faculties training the types of professionals needed by our 'aging society'?^4^. Is this the time to change the model of hospital care centered on acute care and go on to a model aimed at the chronic co-morbidity and dependent patient?.

Let us now think from another perspective. Knowing how the family will evolve during the following decades^5^, which in our countries is the first provider of care for the elderly, will allow us to anticipate aspects related to healthcare attention and provision. How will this affect the labor market, migration of healthcare professionals and individuals from the rural setting to the cities, patterns of education and others to our societies, given that our patients and ourselves live there.

Aging, therefore, will condition the whole society. We must assume it and consider it as a high social responsibility challenge and determinant economic consequences that will affect us in how to conduct our daily medical practice at all levels. This will not find solutions from a given Government Ministry or institution; an inclusive and supportive commitment is required from the public authority, civil society, and the private sector. This is a vision in which solidarity will play a decisive role.

In our case as physicians, health care, attention, and planning tells us of the importance of differentiating between a problem and a necessity. If there is a challenge in healthcare and social protection it is aging and its consequences. We are up against a new challenge with new needs to solve. The necessity in contrast to a problem expresses a difference with the optimum state, i.e., with which we seek to solve. A healthcare need provokes a need for services.

Thinking how our profession will meet these new demands requires reflection and dialogue. A setting where the search for solutions must prevail and not the classifications we use for our comfort. Today, Latin America is experiencing its demographic "moment of opportunity"; it is still a Region with a young mean age. But every moment of opportunity passes, hopefully as physicians we will not let our moment of opportunity pass, for the benefit of our patients and the next generations of older adults, who in 2012 have another cause for celebration - their "own" World Health Day. 
